# Evaluation of jaw bone changes in patients with asthma using inhaled corticosteroids with mandibular radiomorphometric indices on dental panoramic radiographs

**DOI:** 10.4317/medoral.25722

**Published:** 2023-01-15

**Authors:** Sevcihan Günen-Yılmaz, Zeliha Aytekin

**Affiliations:** 1 ORCID: 0000-0002-4566-2927. DDS, PhD. Department of Oral and Maxillofacial Radiology, Faculty of Dentistry, Akdeniz University, Antalya, Turkey; 2 ORCID: 0000-0002-6743-1994. DDS, PhD. Department of Periodontology, Faculty of Dentistry, Akdeniz University, 07058, Antalya, Turkey

## Abstract

**Background:**

Inhaled corticosteroids (ICSs) are an effective drug commonly used in asthma treatment. It is known that osteoporotic changes can occur secondary to steroid usage, depending on dosage and duration. The aim of this study was to compare radiomorphometric indices and fractal dimension on panoramic images of patients with asthma using ICSs and healthy controls.

**Material and Methods:**

A total of 66 dental panoramic radiographs (DPRs) taken from 32 patients with asthma using ICSs and 34 healthy individuals were evaluated in this retrospective study. Panoramic mandibular index inferior and superior (PMI-i,PMI-s), mandibular cortical width (MCW), gonial index (GI), antegonial index (AI), mandibular cortical index (MCI), and fractal dimension analysis (FDA) were measured on DPRs.

**Results:**

PMI-s (*p*=0.02), MCW (*p*<0.001), GI (*p*<0.001) and AI (*p*<0.001) values were significantly lower in the group of the asthma using ICSs than control group. However, the PMI-i (*p* ˃0.05) measurement, the MCI (*p* ˃0.05) and FDA values distribution were similar in both groups.

**Conclusions:**

The use of ICSs in asthma patients can affect bone quality. The evaluation of PMI-s, MCW, GI, and AI on DPR can help determine the effect of this drug on the jawbones in the early period and select dental and surgical treatment plans appropriately.

** Key words:**Asthma, corticosteroids, osteoporosis, radiomorphometric indices, fractal dimension.

## Introduction

Asthma is a respiratory disease characterized by reversible airflow obstruction with airway inflammation and hypersensitivity. Asthma is a common chronic disease affecting from 1% to 20% of the population depending on the country and an estimated 300 million people worldwide. Although it is more common in women and individuals of low socioeconomic status, asthma can affect people of all ages and has a high mortality rate in elderly individuals (1).

Inhaled corticosteroids (ICSs) are the most effective anti-inflammatory drug used in the treatment of some chronic obstructive pulmonary diseases and asthma. ICSs improve lung function, symptoms and quality of life as well as reduce exacerbations and mortality in patients with asthma (2). Long-term use of ICSs may cause systemic side effects, which occur as a result of disruption of the hypothalamus-pituitary-adrenal conduction interaction and include cataracts, Cushing's syndrome, adrenal insufficiency, osteoporosis, dermal thinning and bruising, and growth suppression in children (2). ICS-induced suppression of the adrenal gland leads to reductions in bone mineral density (BMD), causing osteopenia, osteoporosis and osteonecrosis, particularly with long-term and high-dose use. ICSs also have deleterious effects on the function and lifespan of osteoblasts and osteocytes and are known to induce metabolic bone disease by prolonging osteoclast survival (2). ICSs are associated with reductions in BMD. Detection of low BMD is an indicator of osteoporotic changes in bone (3).

The most effective method for determining low BMD is dual-energy X-ray absorptiometry (DEXA). It is an expensive measurement tool that is easy to use in the clinic but not easily accessible for all patients. In addition, the most important disadvantages of the DEXA method are that it cannot distinguish between cortical and trabecular bone and it can elicit false-positive results (4). Dental panoramic radiographs (DPRs), on the other hand, are widely used in dental practice and can safely determine BMD and evaluate osteoporotic changes (5). Mandibular bone mass and the microarchitecture of cortical and trabecular bone can be evaluated using various mandibular indices on DPR, enabling the assessment of osteopenia and osteoporosis (6).

Fractal dimension analysis (FDA), a statistical tissue analysis and it can be used to evaluate the microarchitecture of the mandibular bone mass and trabecular bone (6). Although FDA was used in many diseases or conditions that may cause osteopenia and osteoporosis in some studies in the literature before (6), to the best of our knowledge there was no study investigating mandibular bone morphology in patients with asthma using ICSs by radiomorphometric indices and FDA analysis on DPRs.

The present study aimed to evaluate the effects of ICSs commonly used in asthma treatment on BMD of mandibular bone using radiomorphometric measurements and FDA performed on DPRs.

Materials and Methods

- Study Design

In the study, subjects who had applied to the Oral and Maxillofacial Radiology Department of Akdeniz University, Faculty of Dentistry between January 2017 and December 2018 for various reasons and underwent DPR for any reason were retrospectively evaluated. The study of two groups: the patients with asthma using ICSs and the healthy control group. A total of 66 dental panoramic radiographs (DPRs) taken from 32 patients with asthma using ICSs and 34 healthy individuals were evaluated in this retrospective study. This study was limited to individuals between the ages of 18 and 45 years. Because endocrine changes specific to the onset of perimenopause have been shown to begin near the age of 45, this age was considered the age cut-off point.

The patient group subjects were formed from those who use ICSs twice a day for maintenance treatment for at least one year according to the previous medical records and who did not have an acute asthma attack for the last one year.

Inclusion criteria for the study group were the absence of any systemic disease, pathology, or drug use that could affect bone metabolism except asthma in the patients in the entire study group, and the absence of any disease, including asthma, in the control group. Radiographs of adequate radiographic quality were selected and the inferior mandibular border and one or both mental foramen should be visible on the DPR.

Exclusion criteria for the study group were intrinsic artifacts and patient positioning errors on DPR, history of diseases that affect bone metabolism, history of malignancy or a benign lesion in the mandible, a documented history of consumption of any drugs affecting bone metabolism, history of maxillofacial trauma, and history of pathological fracture.

- Obtaining Radiographs

All DPRs had been obtained using the same panoramic devices (Planmeca Oy, Helsinki, Finland) with the same exposure parameters: (66 kVp, 7 mA, and 16 s exposure time). The Frankfurt plane of the patients was positioned parallel to the floor and the right-hand plane was parallel to the vertical plane of the device. All dental panoramic radiographs were recorded in DICOM image processing software.

- Radiomorphometric Measurements

**Figure 1:**
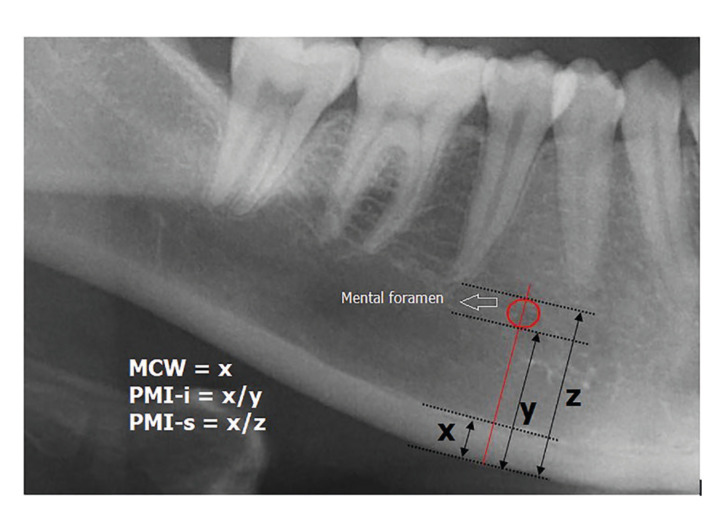
Panoramic radiograph illustrating the measurement of the mandibular cortical width (MCW) and the inferior panoramic mandibular index (PMI-i) and superior panoramic mandibular index. MCW is defined as x. The inferior PMI is defined as the ratio of x/y. The superior PMI is defined as the ratio of x/z. x: Mandibular cortical width; y, Distance from the inferior border of the mandible to the inferior margin of the mental foramen z: Distance from the superior border of the mandible to the inferior margin of the mental foramen.

**Figure 2:**
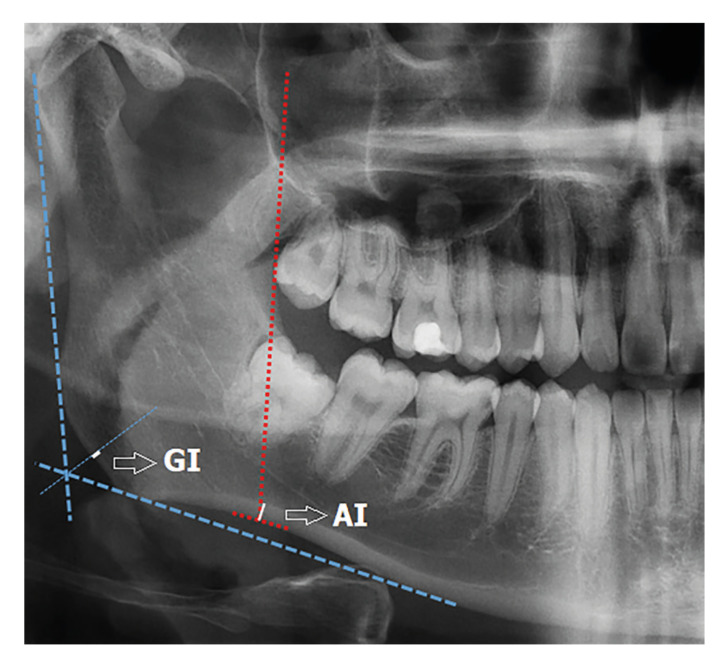
Panoramic radiograph illustrating the measurement of the antegonial index (AI) and gonial index (GI). AI is defined as the mandibular cortical width measured at the intersection of a tangent line to the anterior border of the ramus with the straight line along the inferior mandibular border. GI is defined as the measurement of the mandibular cortical thickness in the area defined by the straight line extending tangentially from the anterior border of the ascending ramus to the inferior border of the mandible.

Panoramic mandibular index (PMI), mandibular cortical width (MCW), GI (Gonial index), antegonial index (AI), mandibular cortical index (MCI), and FDA were measured on DPRs.Inferior panoramic mandibular index (PMI-i) and superior panoramic mandibular index (PMI-s) were calculated as the ratio of the mandibular cortical thickness in the mental region over the distance between the lower border of the mandible and either the inferior or the superior border of the mental foramen (7) (Fig. 1). MCW is the cortical bone thickness in the mandibular base, which is located on the line that is perpendicular to the imaginary line passing from the midpoint of the mental foramen and tangent to the mandibular base (8) (Fig. 1). GI is the width of the inferior mandible cortical border at the gonial angle. The gonial angle is defined as the angle of the lines tangent to the posterior border of the ramus and the inferior border of the mandible (9) (Fig. 2). AI is the measurement of the mandibular cortical thickness measured in the area defined by the straight line extending tangentially from the anterior border of the ascending ramus to the inferior border of the mandible (5) (Fig. 2).

**Figure 3:**
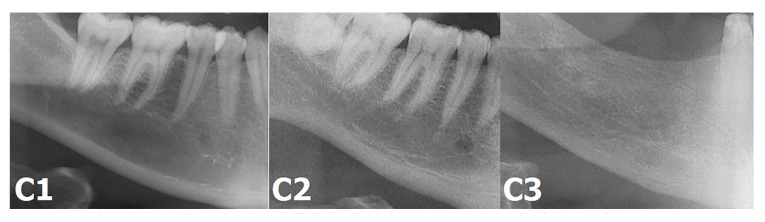
Panoramic radiographs illustrating the mandibular cortical index. C1: Endosteal margin of the cortex is even and sharp; C2: Endosteal margin exhibits semilunar defects or endosteal cortical residues; C3: Cortical layer is clearly porous and with reduced thickness.

MCI is a method based on visual morphological evaluation of the mandibular cortex distal to the mental foramen on DPR. The types of MCI are as: C1, the endosteal margin of the mandibular cortex is continuous and sharp; C2, the endosteal margin of the mandibular cortex appears as semi-lunar defect or endosteal cortical residues; C3, the cortical layer is highly endosteal cortical residues and clearly porous (10) (Fig. 3).

**Figure 4:**
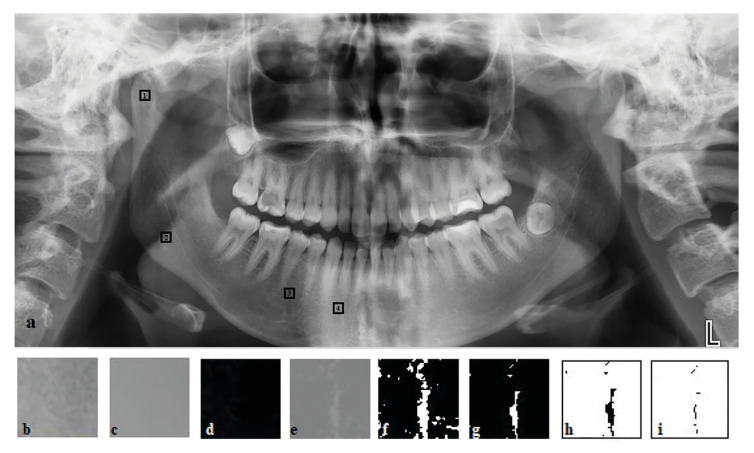
a: Panoramic radiograph with the five selected region of interest areas (ROIs); 1: the subcortical area of the condyle (ROI 1), 2: the supracortical area in the angulus (ROI 2), 3: the mesial area of the first molar (ROI 3), and 4: the apical area of the incisors (ROI 4) ; b: cropped and duplicated image; c: a gaussian blurred image ; d: a subtraction image ; e: an added 128 image ; f: binarization; g: erosion; h: dilation; and i: skeletonization.

For the FDA, all the images were analyzed using ImageJ version 1.3 (National Institutes of Health, Bethesa, MD, USA). ImageJ was used to measure linear distance and calculate fractal dimension by using the box-counting method recommended by White and Rudolph (11). Regions of interest (ROI) were selected from four different 64x64 pixel regions in the right mandible on DPRs. The subcortical area of the condyle (ROI 1), the supracortical area in the angulus (ROI 2), the mesial area of the first molar (ROI 3), and the apical area of the incisors (ROI 4) (Fig. 4).

All DPRs were saved in TIFF format. After ROI was selected, the image was cropped and duplicated. Gaussian filter was applied to the duplicated image. The resulting images were extracted from the original images, and 128 shades of gray were added to each pixel. Bone trabeculae were distinguished to distinctively visualize the spaces between trabeculae. After the images were converted to black and white with the threshold option, the noise was reduced with the ‘erode’ and ‘dilate’ options. After applying the ‘invert’, ‘skeletonize’, and ‘analyze’ functions, FDA was calculated (Fig. 4).

In order to minimize the margin of error for the evaluator, all measurements on the DPRs were evaluated in the early hours of the day and in a dimly lit environment with no more than 10 patients per day. All radiomorphometric measurements were investigated in the DPR of all the subjects by an oral radiologist (S.G.Y.) with 10 years of experience who were blinded to the subject category. The radiomorphometric indices were Figured out bilaterally on the mandibular bone, and mean values were recorded. The measurements were done and the mean value was recorded for statistical analysis for PMI-i, PMI-s, MCW, GI, AI, and FDA. Regarding MCI, the opinion of a more experienced second investigator was sought for DPRs which cannot be decided.

- Evaluation of Intraobserver Reliability

The radiographic measurements were performed by one of the authors. To test within-observer reliability, all measurements on 20 DPRs images (10 DPRs from each group) were re-evaluated one month later. The intra-observer correlation coefficient for qualitative data and kappa statistical tests for categorical data were used (12,13). The criteria were recommended by Koo and Li (12) for interpretation of ICC values of qualitative data. (poor: ˂0.5, fair:0.5-0.75, good:0.75—0.90, excellent: 0.9-1). The criteria were recommended by Landis and Koch (13) for interpretation of kappa values of categorical data (poor <0, slight: 0-0.2, fair: 0.21-0.4, moderate: 0.41-0.6, substantial: 0.61-0.8, or almost perfect 0.81-1)

- Statistical Analysis

All statistical analysis was made using SPSS-version 22 for Windows (IBM Corp., Armonk, NY, USA). Basic descriptive statistical analysis and normality tests of all variables were performed. The normality test of the distribution was evaluated using the Kolmogorov-Smirnov method. Age, GI, AI, and FDA measurements were showed normal distribution but gender, PM-Ii, PMI-s, MCW, and MCI were nonparametrically distributed. To compare the results of the asthma patients using ICSs and healthy controls, Chi-square test was used to evaluate gender and MCI. Age, GI, AI, and FDA measurements distribution between the groups were evaluated with the Student t-test, and PMI-i, PMI-s, and MCW were evaluated with the Mann-Whitney U test. Statistical significance level was considered as the value of *p* ˂ 0.05.

## Results

The mean age of all individuals included in the study was 33.89 ± 9.35 years (min-max age: 18–45 years, *n* = 66), the mean age of the patient group (n = 32) was 32.84 ± 9.11 years and the mean age of the control group was 34.88 ± 9.58 years. The age distribution of both groups was similar, with no statistically significant difference between them (*p* = 0.38) (Table 1).

PMI-i values were higher in the control group than in the patient group, though without a statistically significant difference (*p* = 0.14). PMI-s values were significantly lower in the patient group than in the control group (*p* = 0.02) (Table 1). When the MCW values of the groups were examined, the patient group had a lower mean MCW than the control group, with a statistically significant difference (*p* < 0.001) (Table 1). Both GI and AI measurement values were significantly lower in the patient group than in the control group (*p* < 0.001) (Table 1). There was no statistically significant difference in gender distribution between the patient and control groups (*p* = 0.32). There was no statistically significant difference between the groups in the distribution of MCI types (*p* = 0.24). (Table 2). In all FDA measurements, the patient group was lower than the control group. However, all FD measurements were statistically similar in the control and patient groups (Table 1).

Intraobserver reliability was ranged from good to excellent, for PMI-s, MCW, GI, AI, and FDA measurements. The weighted Cohen kappa coefficient was almost perfect for MCI with a 0.92 value. Intraobserver reliability value was obtained as a result of ICC analysis for qualitative data of radiometric indices are presented in Table 3.

**Table 1:**
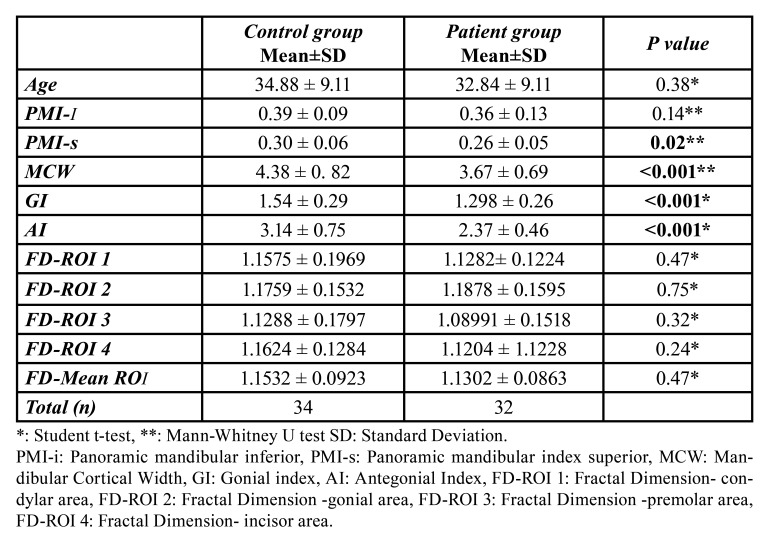
Evaluation of numerical variables between groups.

**Table 2:**
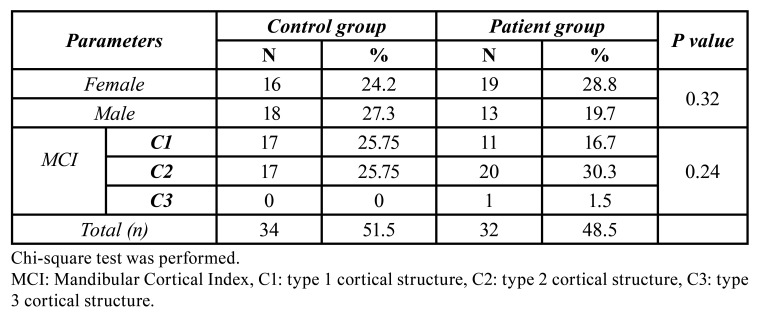
Evaluation of categorical variables between groups.

**Table 3:**
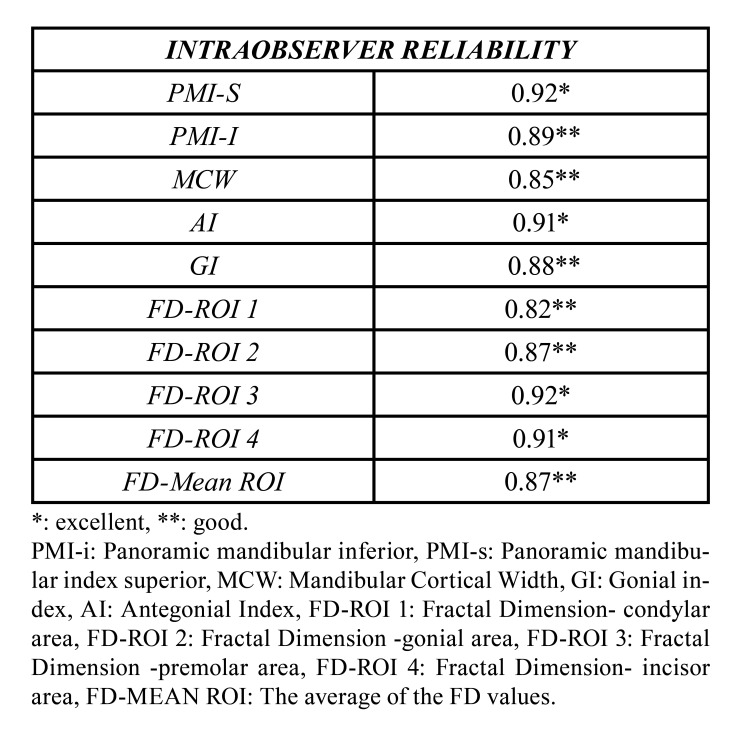
Intraobserver reliability was calculated with the intraclass correlation coefficient for the radiomorphometric indices with quantitative measurements.

## Discussion

This study found significantly lower PMI-s, MCW, GI and AI scores in patients with asthma using ICSs for more than one year compared to healthy controls. These results suggested that ICSs may cause osteoporotic changes in jawbone. While a previous study (14) has evaluated the use of ICSs in patients with asthma in terms of periodontal and dental complications, to the best of our knowledge, this is the first study showing osteoporotic changes using radiomorphometric indices and FDA.

The link between systemic steroids and osteoporosis is well established (15). In a previous study, the effect of systemic corticosteroid use on BMD in the jaw bones was evaluated and it was found that the radiomorphometric index and FDA values were lower in those using systemic corticosteroids than in the healthy group (16). The ICSs have local side effects, such as oropharyngeal candidiasis, hoarseness and cough due to upper respiratory tract irritation (3). In addition, an increase in malocclusion and open bite was found in children using ICSs (17). However there are conflicting studies on the effects of ICSs have on bone health (15). While some studies (2,18) have reported that the use of ICSs reduces bone mineral density and increases the risk of fracture, other study (19) has associated this with the nature of the chronic respiratory disease or patient-related factors (smoking, etc.), not drug use. Since inhaled corticosteroids are administered in an area adjacent to the jawbones, the local side effect on the jawbones may be more serious than in other bones. To the best of the authors’ knowledge, this is the first study in the literature concerning the use of radiomorphometric indices and FDA for evaluating BMD in patients with asthma using ICSs.

Han *et al*. (20), using the DEXA method, reported that 81 individuals between 45 and 70 years of age using ICSs for at least one year demonstrated age-related increases in mandibular osteoporotic changes, which pose a risk for dental problems and losses. The effects on bone metabolism are observed more in patients using medium to high doses of ICS, and these patients should be made aware of the risk of metabolic diseases of the bone (21). Providing these patients with vitamin D and bisphosphonate support and, if possible, reducing the ICS dose may be solutions to reduce the effects on bone (21).

DEXA is the gold standard for examining changes in bone structure in the general population (4). However, evidence indicates that DPR, which is frequently used for imaging purposes in dentistry, is a safe tool for detecting osteoporotic changes in mandibular bone structure using various mandibular indices (22). In addition, DPR is a practical method for evaluating BMD due to easy application and lower radiation exposure (5).

Several studies have evaluated BMD in different conditions and diseases using mandibular radiomorphometric indices (7,11,23). Commonly used radiomorphometric mandibular indices are PMI, MCW, GI, AI and MCI, which, through length and proportional measurements performed on DPRs, provide an indication of the cortical and cancellous structure of the bone (5,22).

The PMI index has been evaluated in many studies to detection of osteoporotic changes. Dagistan *et al*. (22) found a statistically significant difference in osteoporotic individuals. Aktuna Belgin *et al*. (16) found a difference in PMI values between the group using intravenous corticosteroids and the healthy control group, though it was not statistically significant. In the present study, both PMI-i and PMI-s values were lower in the group using ICSs compared to the control group, and the between group difference in PMI-s value was statistically significant.

Dagistan *et al*. (22) reported that the MCW, PMI and AI indices could be used to evaluate osteoporotic changes in both men and women, though MCI was not suiTable. Calciolari *et al*. (24) found that MCI, MCW and PMI were suiTable indices for detecting low BMD. Amed-Al Dam (25) also showed MCI to be a reliable diagnostic tool in elderly patients. Radiomorphometric mandibular indices have been used to evaluate BMD in different conditions and diseases.

In light of the findings of previous studies, a low MCW score has been presented as a reliable indicator of osteoporosis (8,23). Carmo *et al*. (23) found that DPRs could be used to diagnose early mandibular cortical bone density loss and support dental treatment in young postmenopausal women. Some studies have reported lower MCW values in patient groups with compromised bone metabolism, including osteoporotic men aged 45–72 years (22), and patients with chronic renal failure (26), compared to healthy control groups. Aktuna Belgin *et al*. (16) performed a study on patients undergoing intravenous corticosteroid therapy and found significantly lower MCW values in the patient group than in the healthy control group. Consistent with the literature, lower MCW scores were found in the patient group compared to the control group in our study.

In studies comparing patient groups with poorly controlled type 1 diabetes mellitus (27), and chronic renal failure (28) with healthy controls, GI values were significantly lower in the patient groups. However, Yalcin *et al*. (29) found that the GI values of patients with scleroderma were not significantly different than the control group. In line with many previous studies, in the present study, the GI values were found significantly lower in patients using ICSs than in the control group.

AI is the measurement of the cortical width in the region anterior to the gonion at a point identified by extending a line of best fit on the anterior border of the ascending ramus down to the lower border of the mandible. It provides an important panoramic view of osteoporotic changes. Dagistan *et al*. (28) evaluated the mandibular changes of male patients with chronic renal failure and found their AI value to be lower than healthy controls. Similarly, Dutra *et al*. (9) also found AI scores to be significantly different between patient and control groups and correlated AI scores with osteopenia/osteoporosis. Measurement of AI may be a useful method of identifying decreased BMD in groups at high risk for osteoporosis. In the present study, in parallel with previous studies, significantly lower AI values were found in the patient group than in the control group.

MCI calculated on DPRs has been reported in some studies as a repeaTable and reliable indicator of mandibular bone changes and low BMD (10). On the contrary, other studies have not found significant differences in MCI between patients with osteoporotic changes and control groups (28). These inconsistencies in outcomes may be due to differences in patient profiles across the studies. In the current study, MCI was similar in both groups.

In the literature, there are studies in which FDA was used in the evaluation of trabecular bone structure in patients with different systemic diseases (8,11,16,26). While FDA measurements were lower (16,26) or higher (30) in various diseases and conditions compared to the healthy control group, no significant difference was found in some diseases or conditions (30). According to Aktuna Belgin *et al*. (16) presented in their study of the patients receiving intravenous corticosteroid treatment, the FDA values in the condyle, angle of the mandible, and mental foramen region were significantly lower in the systemic corticosteroid group and there was no significant difference in the second premolar area. Contrary to this study, in our study, all FDA values were not statistically different in the patient and control groups. This may be due to the difference in the way ICSs are administered, their mechanism of action, doses, and DPRs devices used.

The present study has several limitations that warrant mentioning. First, it had a single-center, retrospective and cross-sectional design. Cross-sectional investigations lack the power to show true-negative results and bias can be introduced if the case and control groups are not properly matched. Second, the study had a small sample, decreasing the likelihood of finding significant differences and increasing the risk of missing real differences. Third, the DEXA measurement values of the entire study group were not available. Fourth, the osteoportic changes in the mandibular bone could not be evaluated in patient group with asthma who did not use any medication.

## Conclusions

In the present study, it was shown that ICSs cause osteoporotic changes in the mandibular bone. Professionals in dental and oral surgical practices should consider that these side effects of ICSs, which are widely used for the treatment of individuals with asthma, increase the risk for dental health complications.

Future studies evaluating the pre-treatment and post-treatment radiographs of patients with asthma who have received the same dose and duration of ICSs compared with a control group of similar age with no additional systemic diseases can contribute to the literature by supporting our results with clinical and laboratory parameters.

## References

[1] King-Biggs MB (2019). Asthma. Ann Intern Med.

[2] Raissy HH, Kelly HW, Harkins M, Szefler SJ (2013). Inhaled corticosteroids in lung diseases. Am J Respir Crit Care Med.

[3] Velayudhan Nair V, Thomas S, Thomas J, Mathew CM (2017). Panoramic Radiographs for Detecting Osteopenia: A Pilot Study. Clin Pract.

[4] Choël L, Duboeuf F, Bourgeois D, Briguet A, Lissac M (2003). Trabecular alveolar bone in the human mandible: a dual-energy x-ray absorptiometry study. Oral Surg Oral Med Oral Pathol Oral Radiol Endod.

[5] Ledgerton D, Horner K, Devlin H, Worthington H (1997). Panoramic mandibular index as a radiomorphometric tool: an assessment of precision. Dentomaxillofac radiol.

[6] Apolinário AC, Sindeaux R, de Souza Figueiredo PT, Guimarães AT, Acevedo AC, Castro LC (2016). Dental panoramic indices and fractal dimension measurements in osteogenesis imperfecta children under pamidronate treatment. Dentomaxillofac radiol.

[7] Benson BW, Prihoda TJ, Glass BJ (1991). Variations in adult cortical bone mass as measured by a panoramic mandibular index. Oral Surg Oral Med Oral Pathol.

[8] Taguchi A, Sugino N, Miki M, Kozai Y, Mochizuki N, Osanai H (2011). Detecting young Japanese adults with undetected low skeletal bone density using panoramic radiographs. Dentomaxillofac Radiol.

[9] Dutra V, Yang J, Devlin H, Susin C (2005). Radiomorphometric indices and their relation to gender, age, and dental status. Oral Surg Oral Med Oral Pathol Oral Radiol Endod.

[10] Klemetti E, Kolmakov S, Kröger H (1994). Pantomography in assessment of the osteoporosis risk group. Scand J Dent Res.

[11] White SC, Rudolph DJ (1999). Alterations of the trabecular pattern of the jaws in patients with osteoporosis. Oral Surg Oral Med Oral Pathol Oral Radiol Endod.

[12] Koo TK, Li MY (2016). A guideline of selecting and reporting intraclass correlation coefficients for reliability research. J Chiropr Med.

[13] Landis JR, Koch GG (1977). The measurement of observer agreement for categorical data. Biometrics.

[14] Doğan M, Şahiner Ü M, Ataç AS, Ballıkaya E, Soyer Ö U, Şekerel BE (2021). Oral health status of asthmatic children using inhaled corticosteroids. Turk J Pediatr.

[15] Pandya D, Puttanna A, Balagopal V (2014). Systemic effects of inhaled corticosteroids: an overview. Open Respir Med J.

[16] Belgin CA, Serindere G (2020). Fractal and radiomorphometric analysis of mandibular bone changes in patients undergoing intravenous corticosteroid therapy. Oral Surg Oral Med Oral Pathol Oral Radiol.

[17] Kumar S, Nandlal B (2012). Effects of Asthma and Inhalation corticosteroids on the dental arch morphology in children. J Indian Soc Pedod Prev Dent.

[18] Ieda Y, Nagasaka Y (2007). Secondary osteoporosis: inhaled corticosteroids induced osteoporosis in respiratory diseases. Clin Calcium.

[19] Vestergaard P, Rejnmark L, Mosekilde L (2007). Fracture risk in patients with chronic lung diseases treated with bronchodilator drugs and inhaled and oral corticosteroids. Chest.

[20] Han ER, Choi IS, Kim HK, Kang YW, Park JG, Lim JR (2009). Inhaled corticosteroid-related tooth problems in asthmatics. J Asthma.

[21] Xiaomei W, Hang X, Lingling L, Xuejun L (2014). Bone metabolism status and associated risk factors in elderly patients with chronic obstructive pulmonary disease (COPD). Cell Biochem Biophys.

[22] Dagistan S, Bilge O (2010). Comparison of antegonial index, mental index, panoramic mandibular index and mandibular cortical index values in the panoramic radiographs of normal males and male patients with osteoporosis. Dentomaxillofac radiol.

[23] Carmo JZB, Medeiros SFd (2017). Mandibular Inferior Cortex Erosion on Dental Panoramic Radiograph as a Sign of Low BoneMineral Density in Postmenopausal Women. Rev Bras Ginecol Obstet.

[24] Calciolari E, Donos N, Park J, Petrie A, Mardas N (2015). Panoramic measures for oral bone mass in detecting osteoporosis: a systematic review and meta-analysis. J Dent Res.

[25] Al-Dam A, Blake F, Atac A, Amling M, Blessmann M, Assaf A (2013). Mandibular cortical shape index in non-standardised panoramic radiographs for identifying patients with osteoporosis as defined by the German Osteology Organization. J Craniomaxillofac Surg.

[26] Gumussoy I, Miloglu O, Cankaya E, Bayrakdar IS (2016). Fractal properties of the trabecular pattern of the mandible in chronic renal failure. Dentomaxillofac radiol.

[27] Limeira FIR, Rebouças PRM, Diniz DN, Melo DPd, Bento PM (2017). Decrease in mandibular cortical in patients with type 1 diabetes mellitus combined with poor glycemic control. Braz Dent J.

[28] Dagistan S, Miloglu O, Caglayan F (2016). Changes in jawbones of male patients with chronic renal failure on digital panoramic radiographs. Eur J Dent.

[29] Yalcin ED, Avcu N, Uysal S, Arslan U (2019). Evaluation of radiomorphometric indices and bone findings on panoramic images in patients with scleroderma. Oral Surg Oral Med Oral Pathol Oral Radiol.

[30] Kato CN, Barra SG, Tavares NP, Amaral TM, Brasileiro CB, Mesquita RA (2020). Use of fractal analysis in dental images: a systematic review. Dentomaxillofac radiol.

